# The use of volumetric analysis to improve cardiac magnetic resonance evaluation of left ventricular size and function

**DOI:** 10.1186/1532-429X-11-S1-P223

**Published:** 2009-01-28

**Authors:** Victor Mor-Avi, Roberto M Lang, Johannes Niel, Regina Steringer-Macherbauer, Lynn Weinert, Lissa Sugeng, Rolf Baumann, Georg Schummers, Amit Patel, Hans-Joachim Nesser

**Affiliations:** 1 University of Chicago, Chicago, IL USA; 2 Public Hospital Elisabethinen, Linz, Austria; 3 TomTec Imaging Systems, Unterschleissheim, Germany

**Keywords:** Cardiac Magnetic Resonance, Cardiac Magnetic Resonance Image, Left Ventricular Volume, Volumetric Analysis, Volumetric Approach

## Introduction

Cardiac magnetic resonance (CMR) imaging is the current standard reference technique for left ventricular (LV) volume measurements, which are obtained from short-axis slices using the method of disks (MOD) approximation. As new three-dimensional (3D) imaging and volumetric analysis techniques are developed and compared against this reference, there is growing evidence that the standard MOD-based CMR reference has limitations. Thus, the recently developed real-time 3D echocardiography was found to consistently underestimate LV volumes, among other reasons because of CMR errors at the LV base, associated with the use of frequently oblique short-axis views

## Purpose

The aim of this study was to test a new technique for volumetric analysis of CMR images that is free of these errors by comparing it to the standard MOD-based analysis.

## Methods

Steady-state free precession dynamic gradient-echo images (Siemens MAGNETOM Sonata 1.5 T scanner) were obtained in 45 patients with a wide range of LV size and function in short-axis views from LV base to apex as well as 3 long-axis planes rotated around the LV axis. Images were analyzed using the standard MOD technique and, independently, using the new volumetric approach implemented in prototype analysis software (TomTec Imaging Systems). This analysis is based on semi-automated reconstruction of endocardial surface from a combination of short- and long-axis CMR images, followed by direct quantification of LV volume confined within the endocardial surface. Both analysis techniques were used to obtain LV end-systolic and end-diastolic volumes (ESV, EDV) and ejection fraction (EF). Inter-technique comparisons included linear regression and Bland-Altman analyses. Reproducibility of both techniques was assessed in a subset of 10 patients using blinded repeated measurements by the same observer one week later, as well by a second independent observer.

## Results

Despite the high correlation between the two CMR techniques (ESV: r = 0.99; EDV: r = 0.98; EF: r = 0.92), the volumetric approach resulted in significantly smaller volumes, compared to the standard MOD technique (inter-technique differences: ΔESV = 17 ± 14 ml (p < 0.05); ΔEDV = 20 ± 16 ml (p < 0.05); ΔEF = 2 ± 6%; (NS). Intra- and inter-observer variability of the MOD technique for both volumes and EF was 2 to 7% of the mean measured value, while that of the volumetric approach was 5 to 10%, probably reflecting the users' learning curve with the new technique.

## Conclusion

**Figure 1:**
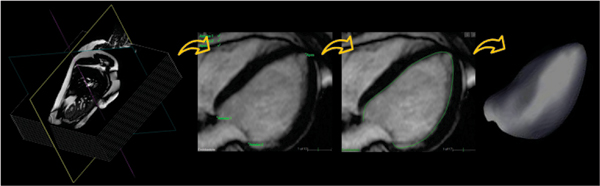
Figure 1

Volumetric analysis of CMR images, which does not rely on criteria for basal LV slice selection, results in smaller LV volume measurements. With its relatively high reproducibility, this approach may provide an alternative, potentially more accurate CMR reference technique for LV volume measurements, to which other 3D imaging modalities would be compared more fairly (Figure 1).

